# [Bis(2-pyridylmeth­yl)amine]dichloridomercury(II)

**DOI:** 10.1107/S1600536808000858

**Published:** 2008-01-16

**Authors:** Young-Inn Kim, You-Soon Lee, Hoe-Joo Seo, Ki-Sun Nam, Sung Kwon Kang

**Affiliations:** a Department of Chemistry Education and Center for Plastic Information Systems, Pusan National University, Pusan 609-735, Republic of Korea; b Department of Chemistry, Chungnam National University, Daejeon 305-764, Republic of Korea; c Department of Chemistry, Pusan National University, Pusan 609-735, Republic of Korea; d Interdisciplinary Program of Advanced Materials for Information and Display, Pusan National University, Pusan 609-735, Republic of Korea

## Abstract

The Hg atom in the title complex, [HgCl_2_(C_12_H_13_N_3_)], adopts a square-pyramidal geometry, being ligated by three N atoms of the tridentate bis­(2-pyridylmeth­yl)amine ligand and two Cl atoms, with one of the latter occupying the apical position. Disorder is noted in the amine portion of the ligand and this was modelled over two sites, with the major component having a site-occupancy factor of 0.794 (14).

## Related literature

For general background, see: Ojida *et al.* (2004 ▶); Kirin *et al.* (2005 ▶); Storr *et al.* (2005 ▶); Tamamura *et al.* (2006 ▶); Kim *et al.* (2007 ▶); Lee *et al.* (2007 ▶). For related literature, see: Addison *et al.* (1984 ▶).
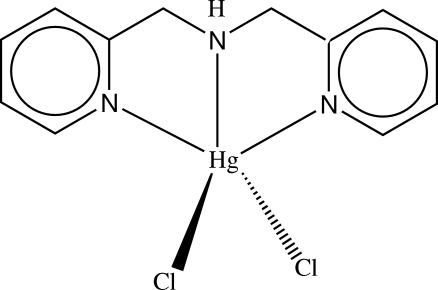


## Experimental

### 

#### Crystal data

[HgCl_2_(C_12_H_13_N_3_)]
*M* *_r_* = 470.74
Monoclinic, 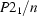

*a* = 8.4083 (6) Å
*b* = 12.8278 (11) Å
*c* = 13.3457 (12) Å
β = 90.462 (2)°
*V* = 1439.4 (2) Å^3^
*Z* = 4
Mo *K*α radiation
μ = 11.05 mm^−1^
*T* = 295 (2) K
0.18 × 0.15 × 0.15 mm

#### Data collection

Bruker SMART CCD area-detector diffractometer
Absorption correction: multi-scan (*SADABS*; Bruker, 2002 ▶) *T*_min_ = 0.143, *T*_max_ = 0.185
15603 measured reflections
3580 independent reflections
2547 reflections with *I* > 2σ(*I*)
*R*_int_ = 0.028

#### Refinement

*R*[*F* ^2^ > 2σ(*F* ^2^)] = 0.027
*wR*(*F* ^2^) = 0.061
*S* = 1.01
3580 reflections
173 parameters
H-atom parameters constrained
Δρ_max_ = 1.22 e Å^−3^
Δρ_min_ = −0.47 e Å^−3^

### 

Data collection: *SMART* (Bruker, 2002 ▶); cell refinement: *SAINT* (Bruker, 2002 ▶); data reduction: *SAINT*; program(s) used to solve structure: *SHELXS97* (Sheldrick, 2008 ▶); program(s) used to refine structure: *SHELXL97* (Sheldrick, 2008 ▶); molecular graphics: *ORTEP-3 for Windows* (Farrugia, 1997 ▶); software used to prepare material for publication: *WinGX* (Farrugia, 1999 ▶).

## Supplementary Material

Crystal structure: contains datablocks global, I. DOI: 10.1107/S1600536808000858/tk2241sup1.cif

Structure factors: contains datablocks I. DOI: 10.1107/S1600536808000858/tk2241Isup2.hkl

Additional supplementary materials:  crystallographic information; 3D view; checkCIF report

## Figures and Tables

**Table:** 

Hg—Cl1	2.4336 (12)
Hg—Cl2	2.4579 (14)
Hg—N1	2.394 (4)
Hg—N8	2.445 (5)
Hg—N15	2.405 (4)

**Table:** 

Cl1—Hg—Cl2	118.63 (5)
N1—Hg—N8	67.82 (15)
N1—Hg—N15	133.99 (12)
N8—Hg—N15	68.04 (14)

**Fig. 1.**
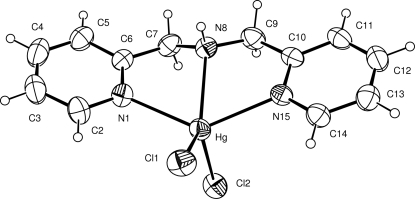
Molecular structure of (I), showing the atom-numbering scheme and 30% probability ellipsoids. Only the major component of the disordered atoms is shown for clarity.

## supplementary crystallographic information

### Comment

Transition metal complexes with bis(2-pyridylmethyl)amine (dpa) or substituted-dpa ligands continue to be of interest in many fields of chemistry (Kirin *et al.*, 2005; Storr *et al.*, 2005; Tamamura *et al.*, 2006). Recently, we reported Cu(II) (Lee *et al.*, 2007) and Zn(II) (Kim *et al.*, 2007) halide complexes with the dpa ligand, and Zn(dpa)Cl_2_ was proposed as a blue fluorescent material. In this work, as an extension of a study on fluorescent chemosensors (Ojida *et al.*, 2004), we prepared a Hg(II) complex of dpa, Hg(dpa)Cl_2_ (I), and its structure and properties were investigated. The Hg(II) atom is 5-coordinated by the three N atoms of the tridentate di(picolyl)amine ligand and two Cl atoms. The coordination geometry is based on a square pyramid with the basal plane defined by three N atoms and one Cl, with the other Cl atom occupying the apical position. The calculated trigonality index, τ = 0.03, indicates that the Hg atom is in a square pyramidal geometry (Addison *et al.*, 1984). Hg(dpa)Cl_2_ exhibits an intense blue emission at 425 nm in DMF solution upon excitation at 400 nm.

### Experimental

All of the reagents and solvents were purchased from either Aldrich and used without further purification. A mixture of mercuric chloride (1.35 g, 5 mmol) and bis(2-pyridylmethyl)amine (0.99 g, 5 mmol) in methanol (20 ml) was stirred for 8 h at room temperature under a nitrogen atmosphere. The precipitates were filtered off and recrystallized from methanol in a 63% yield. ^1^H-NMR for dpa in (I) (d_6_-DMSO, p.p.m.): δ: 8.51 (d, 2H), 7.96 (t, 2H), 7.52 (m, 4H), 4.98 (s, 1H), 4.08 (s, 4H).

### Refinement

The C and N-bound H atoms were included in the riding model approximation with C—H = 0.93–0.97 Å and N—H = 0.91 Å, and with *U*_iso_(H) = 1.2*U*_eq_(C and N). Disorder was noted in the structure and this modelled so that two sites were resolved for the N8—H atoms. From refinement, the major component of the disorder had a site occupancy factor = 0.794 (14). The maximum and minimum residual electron denisty peaks were located 0.85 and 0.78 Å, respectively, from the Hg atom.

### Crystal data

**Table:**

[HgCl_2_(C_12_H_13_N_3_)]	*F*_000_ = 880
*M**_r_* = 470.74	*D*_x_ = 2.172 Mg m^−^^3^
Monoclinic, *P*2_1_/*n*	Mo *K*α radiation λ = 0.71073 Å
Hall symbol: -P 2yn	Cell parameters from 4387 reflections
*a* = 8.4083 (6) Å	θ = 2.2–24.5º
*b* = 12.8278 (11) Å	µ = 11.05 mm^−^^1^
*c* = 13.3457 (12) Å	*T* = 295 (2) K
β = 90.462 (2)º	Block, orange
*V* = 1439.4 (2) Å^3^	0.18 × 0.15 × 0.15 mm
*Z* = 4	

### Data collection

**Table:**

Bruker SMART CCD area-detector diffractometer	*R*_int_ = 0.028
φ and ω scans	θ_max_ = 28.3º
Absorption correction: multi-scan(SADABS; Bruker, 2002)	θ_min_ = 2.2º
*T*_min_ = 0.143, *T*_max_ = 0.185	*h* = −11→11
15603 measured reflections	*k* = −12→17
3580 independent reflections	*l* = −17→17
2547 reflections with *I* > 2σ(*I*)	

### Refinement

**Table:**

Refinement on *F*^2^	H-atom parameters constrained
Least-squares matrix: full	*w* = 1/[σ^2^(*F*_o_^2^) + (0.0268*P*)^2^ + 0.7364*P*] where *P* = (*F*_o_^2^ + 2*F*_c_^2^)/3
*R*[*F*^2^ > 2σ(*F*^2^)] = 0.027	(Δ/σ)_max_ = 0.002
*wR*(*F*^2^) = 0.061	Δρ_max_ = 1.22 e Å^−^^3^
*S* = 1.02	Δρ_min_ = −0.47 e Å^−^^3^
3580 reflections	Extinction correction: none
173 parameters	

### Special details

**Table:**

Geometry. All e.s.d.'s (except the e.s.d. in the dihedral angle between two l.s. planes) are estimated using the full covariance matrix. The cell e.s.d.'s are taken into account individually in the estimation of e.s.d.'s in distances, angles and torsion angles; correlations between e.s.d.'s in cell parameters are only used when they are defined by crystal symmetry. An approximate (isotropic) treatment of cell e.s.d.'s is used for estimating e.s.d.'s involving l.s. planes.

### Fractional atomic coordinates and isotropic or equivalent isotropic displacement parameters (Å^2^)

**Table:**

	*x*	*y*	*z*	*U*_iso_*/*U*_eq_	Occ. (<1)
Hg	0.87912 (2)	0.305769 (14)	0.873548 (12)	0.06174 (8)	
Cl1	1.04268 (15)	0.36276 (12)	0.73426 (9)	0.0791 (3)	
Cl2	0.63148 (16)	0.21091 (10)	0.83674 (11)	0.0794 (4)	
N1	1.0536 (5)	0.1777 (3)	0.9449 (3)	0.0636 (10)	
C2	1.1638 (6)	0.1270 (4)	0.8923 (4)	0.0799 (14)	
H2	1.1708	0.1399	0.8239	0.096*	
C3	1.2665 (7)	0.0573 (5)	0.9348 (5)	0.0911 (17)	
H3	1.3425	0.0235	0.8963	0.109*	
C4	1.2548 (7)	0.0381 (5)	1.0354 (6)	0.0959 (18)	
H4	1.3227	−0.0093	1.0665	0.115*	
C5	1.1423 (6)	0.0898 (4)	1.0893 (4)	0.0792 (14)	
H5	1.1332	0.0779	1.1578	0.095*	
C6	1.0426 (5)	0.1592 (3)	1.0422 (3)	0.0576 (10)	
C7	0.9172 (6)	0.2192 (4)	1.0981 (4)	0.0727 (13)	
H7A	0.815	0.1847	1.0914	0.087*	
H7B	0.9447	0.2228	1.1688	0.087*	
N8	0.9085 (9)	0.3260 (5)	1.0549 (3)	0.0594 (19)	0.794 (14)
H8	1.0033	0.358	1.0671	0.071*	0.794 (14)
N8A	0.807 (3)	0.2914 (15)	1.0593 (11)	0.048 (7)	0.206 (14)
H8A	0.7101	0.2599	1.0615	0.057*	0.206 (14)
C9	0.7863 (6)	0.3898 (4)	1.0952 (3)	0.0746 (14)	
H9A	0.8204	0.4161	1.1601	0.09*	
H9B	0.6918	0.3478	1.1053	0.09*	
C10	0.7454 (5)	0.4802 (4)	1.0282 (3)	0.0590 (11)	
C11	0.6733 (5)	0.5697 (4)	1.0649 (4)	0.0707 (13)	
H11	0.6523	0.5763	1.1329	0.085*	
C12	0.6337 (6)	0.6482 (4)	0.9995 (4)	0.0766 (14)	
H12	0.5842	0.7081	1.0229	0.092*	
C13	0.6666 (6)	0.6383 (4)	0.9010 (4)	0.0742 (13)	
H13	0.6408	0.6912	0.856	0.089*	
C14	0.7391 (5)	0.5482 (4)	0.8685 (3)	0.0669 (12)	
H14	0.7622	0.5413	0.8007	0.08*	
N15	0.7774 (4)	0.4704 (3)	0.9307 (2)	0.0563 (8)	

### Atomic displacement parameters (Å^2^)

**Table:**

	*U*^11^	*U*^22^	*U*^33^	*U*^12^	*U*^13^	*U*^23^
Hg	0.07430 (13)	0.06556 (13)	0.04548 (10)	0.01047 (9)	0.00744 (7)	0.00080 (8)
Cl1	0.0815 (8)	0.0984 (10)	0.0575 (6)	−0.0078 (7)	0.0157 (6)	0.0109 (6)
Cl2	0.0726 (8)	0.0803 (9)	0.0857 (9)	−0.0067 (6)	0.0138 (6)	−0.0032 (6)
N1	0.061 (2)	0.064 (2)	0.066 (2)	0.0019 (18)	0.0042 (18)	0.0070 (18)
C2	0.078 (3)	0.077 (4)	0.085 (3)	0.010 (3)	0.020 (3)	−0.004 (3)
C3	0.074 (4)	0.069 (4)	0.130 (5)	0.010 (3)	0.010 (3)	−0.020 (4)
C4	0.075 (4)	0.072 (4)	0.140 (6)	0.014 (3)	−0.019 (4)	0.006 (4)
C5	0.077 (3)	0.076 (3)	0.084 (3)	0.000 (3)	−0.009 (3)	0.017 (3)
C6	0.056 (3)	0.053 (2)	0.064 (3)	−0.007 (2)	−0.004 (2)	0.008 (2)
C7	0.073 (3)	0.086 (4)	0.059 (3)	0.001 (3)	0.000 (2)	0.017 (3)
N8	0.051 (4)	0.067 (4)	0.060 (3)	−0.003 (3)	0.004 (2)	0.003 (2)
N8A	0.046 (14)	0.053 (12)	0.045 (9)	−0.007 (9)	−0.002 (7)	0.009 (7)
C9	0.089 (4)	0.082 (4)	0.053 (3)	0.000 (3)	0.011 (2)	−0.003 (2)
C10	0.058 (3)	0.063 (3)	0.056 (2)	−0.009 (2)	0.0045 (19)	−0.012 (2)
C11	0.068 (3)	0.076 (3)	0.068 (3)	−0.005 (3)	0.010 (2)	−0.022 (3)
C12	0.058 (3)	0.062 (3)	0.109 (4)	0.002 (2)	−0.001 (3)	−0.021 (3)
C13	0.068 (3)	0.064 (3)	0.090 (4)	−0.003 (3)	−0.003 (3)	0.003 (3)
C14	0.074 (3)	0.061 (3)	0.066 (3)	−0.006 (2)	0.006 (2)	−0.001 (2)
N15	0.060 (2)	0.058 (2)	0.0507 (19)	−0.0047 (17)	0.0061 (16)	−0.0016 (16)

### Geometric parameters (Å, °)

**Table:**

Hg—Cl1	2.4336 (12)	C7—H7A	0.97
Hg—Cl2	2.4579 (14)	C7—H7B	0.97
Hg—N1	2.394 (4)	N8—C9	1.423 (7)
Hg—N8	2.445 (5)	N8—H8	0.91
Hg—N15	2.405 (4)	N8A—C9	1.362 (18)
Hg—N8A	2.563 (17)	N8A—H8A	0.91
N1—C6	1.325 (6)	C9—C10	1.503 (7)
N1—C2	1.336 (6)	C9—H9A	0.97
C2—C3	1.364 (7)	C9—H9B	0.97
C2—H2	0.93	C10—N15	1.336 (5)
C3—C4	1.370 (8)	C10—C11	1.389 (6)
C3—H3	0.93	C11—C12	1.372 (7)
C4—C5	1.365 (8)	C11—H11	0.93
C4—H4	0.93	C12—C13	1.351 (7)
C5—C6	1.372 (6)	C12—H12	0.93
C5—H5	0.93	C13—C14	1.379 (7)
C6—C7	1.508 (7)	C13—H13	0.93
C7—N8A	1.407 (18)	C14—N15	1.337 (6)
C7—N8	1.488 (7)	C14—H14	0.93
			
Cl1—Hg—Cl2	118.63 (5)	H7A—C7—H7B	108.4
N1—Hg—N8	67.82 (15)	C9—N8—C7	114.6 (5)
N1—Hg—N15	133.99 (12)	C9—N8—Hg	111.6 (3)
N8—Hg—N15	68.04 (14)	C7—N8—Hg	106.9 (3)
N1—Hg—Cl1	99.30 (10)	C9—N8—H8	107.8
N15—Hg—Cl1	100.55 (9)	C7—N8—H8	107.8
Cl1—Hg—N8	132.16 (19)	Hg—N8—H8	107.8
N1—Hg—Cl2	104.77 (10)	C9—N8A—C7	124.5 (15)
N15—Hg—Cl2	101.26 (9)	C9—N8A—Hg	107.8 (10)
N8—Hg—Cl2	109.21 (19)	C7—N8A—Hg	104.1 (10)
N1—Hg—N8A	73.5 (4)	C9—N8A—H8A	106.4
N15—Hg—N8A	70.7 (4)	C7—N8A—H8A	106.4
Cl1—Hg—N8A	154.1 (6)	Hg—N8A—H8A	106.4
N8—Hg—N8A	22.0 (5)	N8A—C9—C10	122.3 (7)
Cl2—Hg—N8A	87.2 (6)	N8—C9—C10	112.4 (4)
C6—N1—C2	118.8 (4)	N8A—C9—H9A	126.8
C6—N1—Hg	117.8 (3)	N8—C9—H9A	109.1
C2—N1—Hg	123.4 (3)	C10—C9—H9A	109.1
N1—C2—C3	122.7 (5)	N8—C9—H9B	109.1
N1—C2—H2	118.6	C10—C9—H9B	109.1
C3—C2—H2	118.6	H9A—C9—H9B	107.9
C2—C3—C4	118.4 (5)	N15—C10—C11	120.9 (5)
C2—C3—H3	120.8	N15—C10—C9	117.4 (4)
C4—C3—H3	120.8	C11—C10—C9	121.7 (4)
C5—C4—C3	119.1 (5)	C12—C11—C10	119.1 (5)
C5—C4—H4	120.5	C12—C11—H11	120.5
C3—C4—H4	120.5	C10—C11—H11	120.5
C4—C5—C6	119.8 (5)	C13—C12—C11	120.0 (5)
C4—C5—H5	120.1	C13—C12—H12	120
C6—C5—H5	120.1	C11—C12—H12	120
N1—C6—C5	121.3 (5)	C12—C13—C14	118.6 (5)
N1—C6—C7	116.6 (4)	C12—C13—H13	120.7
C5—C6—C7	122.1 (4)	C14—C13—H13	120.7
N8A—C7—C6	127.9 (8)	N15—C14—C13	122.4 (4)
N8—C7—C6	108.1 (4)	N15—C14—H14	118.8
N8—C7—H7A	110.1	C13—C14—H14	118.8
C6—C7—H7A	110.1	C10—N15—C14	119.0 (4)
N8A—C7—H7B	118.6	C10—N15—Hg	117.8 (3)
N8—C7—H7B	110.1	C14—N15—Hg	122.9 (3)
C6—C7—H7B	110.1		
